# Utilizing triethylenetetramine-functionalized MIP-206 for highly efficient removal of Pb(II) from wastewater

**DOI:** 10.1038/s41598-024-66358-6

**Published:** 2024-07-06

**Authors:** Bizhan Shilani, Reza Mehdipour, Behnam Mousazadeh, Yasin Noruzi, Seyednooroldin Hosseini, Hasan N. Al-Saedi, Sura Mohammad Mohealdeen

**Affiliations:** 1 Department of Petroleum Engineering, EOR Research Center, Omidiyeh Branch, Islamic Azad University, Omidiyeh, Iran; 2 Research and Development Department, Hetcochem Company, Tabriz, Iran; 3 Department of Petroleum Engineering, Amirkabir University, Tehran, Iran; 4 Department of Petroleum Engineering, Al-Amarah University Collage, Missan, Iraq; 5 Department of Radiology & Sonar Technologies, Health and Medical Techniques Collage, Al-Noor University, Mosul, Iraq

**Keywords:** Adsorption, Pb removal, Metal organic framework (MOF), MIP-206-OH, Chemistry, Engineering, Materials science

## Abstract

The global concern over heavy metal pollution necessitates urgent measures to safeguard human health and the environment. This study focuses on employing triethylenetetramine (TETA)-functionalized MIP-206-OH (TMIP-206) as an effective adsorbent for removing Pb(II) from wastewater. TMIP-206 was synthesized via a hydrothermal method followed by functionalization with TETA. Kinetic studies demonstrate that lead removal on TMIP-206 conforms to the pseudo-second-order model, indicating an efficient removal process. Experimental results reveal that TMIP-206 aligns with the Langmuir isotherm, exhibiting a maximum removal capacity of 267.15 mg/g for lead ions. The sorption efficiency of TMIP-206 for Pb ions remains stable across six cycles, with a reduction of less than 15%. Optimal adsorption performance is observed at a pH of 6. These findings underscore the potential of TMIP-206 as an alternative for adsorbing Pb(II) from aqueous environments, addressing the global challenge of heavy metal pollution. Future research should explore the scalability and long-term stability of TMIP-206-based adsorbents to enhance their practical applicability in diverse environmental contexts and contribute to broader strategies for mitigating heavy metal contamination.

## Introduction

Access to clean freshwater is vital for the health and well-being of humans, animals, and plants, and ensuring its purity is critical for preventing diseases, sustaining agriculture, and supporting biodiversity ^1,2^. Over time, scientists have exhibited increasing apprehension regarding environmental pollution, especially water contamination ^3–7^. Unfortunately, industrialization has significantly contributed to water contamination ^8–10^. Therefore, utilizing renewable energy sources has substantial promise in alleviating environmental pollution ^11–13^.

Heavy metals, a primary source of environment pollution especially water, do not easily undergo biodegradation and typically form complexes with atoms of nitrogen (N), oxygen (O), and sulfur (S) ^14,15^. It is because N and S atoms contain lone pairs of electrons, allowing them to function as Lewis bases. Lewis acids, for instance lead ions capable of accepting electrons, can establish coordinate bonds with the S and N atoms (functioning as Lewis bases) ^16^. This leads to the buildup of these substances in the ecosystem and the human body ^17,18^. Lead ions are recognized as highly toxic pollutants, posing significant risks to various parts of the body ^19–21^. For instance, the acculturation of lead ions in the human body can promote problems in various bodily systems, including brain functions, fundamental cellular processes, reproductive system, and renal function ^22–24^. The removal of heavy metals can significantly contribute to maintaining environmental cleanliness and preventing potential health issues ^25,26^. Until now, numerous researchers have experimented with various approaches to purify water resources, including chemical precipitation, membrane separation, biological treatment, and adsorption ^27–30^. Among the diverse methods for removing contaminants, adsorption is notable for its cost-effectiveness, straightforward operation, selectivity, and high efficiency ^31–33^. For instance, many researchers have shown that membrane filtration suffer from a drawback of fouling and costly process ^34^. Biological treatments have been demonstrated to be insufficient and suffer from a rather slow process ^35^. Also, chemical precipitation lacks the required selectivity for metal removal and requires large consumption of chemicals ^36–38^. Numerous materials have found extensive application in the adsorptive elimination of heavy metals from aqueous sources, including altered activated carbon, carbon nanotubes, zeolites, and metal–organic frameworks (MOFs) ^39–42^. In recent times, there has been significant attention given to exploring the potential of MOFs as adsorbents for pollutants ^42^. MOFs are three-dimensional materials constructed from ligand linkers and metal ions as central components ^43^. Because of their distinctive features, including customizable surface functionalization MOFs, elevated porosity, and extensive surface area serve as exceptional porous frameworks for applications such as gas storage, catalysis, photocatalysis, drug delivery, and adsorption-driven separation processes ^42^. Functional groups like nitro, amine, thiol, and sulfur, incorporating nitrogen (N) and sulfur (S) atoms, exhibit notably efficient interactions with heavy metals, particularly lead ions due to functioning as Lewis bases ^44^. In this context, numerous researchers have recently begun evaluating the side functionalization of MOFs and their effectiveness as adsorbents for heavy metals in aquatic environments. Zheng and team designed nanofibrous membranes composed of polyethersulfone and modified with ionic liquids for the purpose of heavy metal removal. Their study highlighted the potential of these innovative membranes with coordination of nitrogen atoms in addressing heavy metal contamination ^45^. In a separate investigation, Mohammadi and co-authors conducted the synthesis of copper-based metal–organic frameworks (Cu-MOFs) with amine modifications, aiming to removal of lead ions from effluent samples ^44^. The porous structure of the MOF, coupled with amine functionalization, acted as an optimal platform for the effective removal of lead ions ^44^. Ke Wang and colleagues synthesized Zr-MOFs functionalized with -NH_2_ groups and employed them for the first time in adsorbing both Pb and Cd ions ^46^. It is evident that functional groups significantly contribute to augmenting the adsorption capacity in the majority of well-known MOFs used as heavy metal adsorbents. Despite these improvements, the pore network morphology of these MOFs suffers from a narrow pore opening and the positioning of functional groups. The ideal pore framework for heavy metal removal can be homogenous pores with open windows containing active sites facing the pore interior, shaping a cage-like structure with clips facing the cage center to keep metal ions inside. Recently, Wang et al., developed a MOF based on Zr clusters and isophthalic acid (IPA) named MIP-206. MIP-206 exhibited mesopores with open entrance as well as remarkable chemical and thermal stability. However, there was a drawback in synthesizing MIP-206 with amine functionalized IPA leading to non-uniform structural phases. Also, its structural stability in water media has yet to be explored. Having considered the advantages offered by MIP-206, a research avenue opens to explore various applications of this material and its behavior in different environments.

The novelty of this research lies in the functionalization of MIP-206-OH with triethylenetetramine, which has not been previously explored for Pb(II) adsorption. This novel approach enhances the adsorption capacity and reusability of the material, offering a promising solution for mitigating heavy metal pollution in aqueous environments. In this study, an initial implementation of triethylenetetramine on MIP-206 (TMIP-206) was performed to incorporate amine moieties into its pore network. TMIP-206 was applied for the adsorptive elimination of lead ions from wastewater, as a representative of heavy metal ions. The resulting product can capitalize on the benefits derived from the amine functional groups present in triethylenetetramine and the porous crystalline framework of MIP-206. Potential experimental factors affecting adsorption were thoroughly examined. The products were characterized using BET, FTIR, XRD, TGA, and SEM. The water stability of MIP-206 and TMIP-206 was analyzed by monitoring its crystallinity using XRD. Furthermore, laboratory data were analyzed with the widely recognized Freundlich and Langmuir isotherms to comprehend the nature of adsorption.

## Experimental

### Chemical agents

The compounds utilized in both the creation of the adsorbent and the lead removal procedure were sourced from reputable companies such as Merck or Sigma Aldrich. The specific substances employed include 5-Hydroxyisophthalic acid (5-OH-IPA) (Merck), chloroform (CHCl_3_) (Sigma Aldrich), Zirconium oxychloride octahydrate (ZrOCl_2_·8H_2_O) (Merck), Triethylenetetramine (TETA) (Merck), hydrochloric acid (HCl) (Merck), formic acid (FA) (Sigma Aldrich), sodium hydroxide (NaOH) (Merck), acetone (C_3_H_6_O) (Merck), Potassium bromide (KBr) (Merck), and lead nitrate (Pb(NO_3_)_2_) (Merck).

### Methodology for adsorbent preparation

In the experimental procedure, 5-OH-IPA weighing 1.44 g and corresponding to 8.0 mmol was meticulously placed into a 46 mL Teflon reactor. Following this, 10 mL of formic acid (FA) was introduced, initiating a stirring process for a period of 5 min at ambient temperature until the formation of a homogeneous suspension was achieved. Subsequently, ZrOCl_2_·8H_2_O weighing 2.86 g and equating to 12 mmol was introduced to the suspension, and the mixture underwent an additional 10 min of stirring at ambient temperature to ensure the uniform dispersion of reactants.

The ensuing reaction mixture was then hermetically sealed within an autoclave and subjected to a gradual temperature increase, reaching 200°C over a period of 2 h, followed by a sustained maintenance at 200°C for a duration of 20 h. Upon cooling to room temperature, the anticipated product, denoted as MIP-206-OH, was obtained in the form of a filtrate weighing 4 g. This filtrate underwent a purification process involving washing with acetone and subsequent air-drying.

The activation process of MIP-206-OH commenced at 120°C through a vacuum heating procedure extending over 12 h. In a separate phase of the experiment, a precisely calculated quantity of TETA was dissolved in 20 mL of CHCl_3_. Subsequently, 200 mg of the activated MIP-206-OH was introduced into the solution, initiating a stirring period lasting 0.5 h. Following this interaction, the solution underwent a meticulous filtration process and underwent successive washes with CHCl_3_, culminating in thorough drying under vacuum conditions for an additional 12 h.

This activation methodology involving controlled temperature and vacuum conditions serves to enhance the functional properties of MIP-206-OH, rendering it more amenable to subsequent chemical interactions. The incorporation of TETA into the activated MIP-206-OH not only broadens the versatility of the material but also facilitates the development of tailored functionalities for targeted applications. The rigorous washing and drying steps post-incorporation ensure the removal of residual reactants, contributing to the refinement of the synthesized composite material.

### Methodology for lead adsorption

Batch experiments were performed to assess the efficacy of TMIP-206 in removing lead ion from water samples. During these trials, 200 mg/L of TMIP-206 was introduced into 100 mL water samples at pH 6 containing lead concentrations varying from 5 to 100 mg/L. Stirring was maintained for a duration of one day at room temperature (25 °C) to reach adsorption equilibrium. After separation of the adsorbent, The FAAS (Flame Atomic Absorption Spectroscopy) instrument was employed to quantify the residual concentration of Pb(II) in the solution. The evaluation of lead uptake performance was conducted utilizing Eqs. (1) and (2).

1 $${Q}_{e}={(C}_{i}-{C}_{e})\times \frac{V}{m}$$

2 $$\text{Pb ion adsorption \%}={(C}_{i}-{C}_{e})\times \frac{100}{{C}_{i}}$$

In this context, Q_e_ (mg/g) shows the equilibrium lead uptake capacity, C_i_ (mg/L) signifies the initial lead concentration before introducing the adsorbent, C_e_ (mg/L) shows the lead concentration at equilibrium, and m (g) and V (L) correspond to the mass of TMIP-206 and the volume of the water sample, respectively.

### Methodology for lead adsorption isotherms

The examination of isotherms was performed utilizing data obtained from lead uptake experiments involving TMIP-206. The data on lead uptake underwent analysis using the Freundlich, Langmuir, and Temkin equations, and the parameters for fitting were computed. The equations employed for the isothermal investigation are outlined as follows:

3 $${q}_{e}=\frac{{q}_{m}\times {K}_{l}\times {C}_{e}}{1+{C}_{e}\times {K}_{l}}$$

4 $${q}_{e}={K}_{f}{C}_{e}^{1/n}$$

5 $${q}_{e}=\frac{RT}{{b}_{T}} Ln\left({A}_{T}\times {C}_{e}\right)$$

In this context, q_e_ (mg/g) signifies the lead uptake at equilibrium, q_m_ (mg/g) signifies the theoretical optimum value for lead uptake, C_e_ (mg/L) represents the lead uptake at equilibrium, and K_l_ (L/mg) stands for the Langmuir fitting parameter. Additionally, K_f_ (mg/g) and n represent the Freundlich parameters, where A_T_ (L/g), ${b}_{T}$, T (K), and R (8.314 J/mol/K) represent the Temkin isotherm equilibrium binding constant, Temkin isotherm constant, temperature, and universal gas constant, respectively.

The impact of reaction duration was examined through lead uptake experiments utilizing 200 mg/L of TMIP-206, a lead concentration ranging from 5 to 100 mg/L, and a sample volume of 100 mL.

### Methodology for lead adsorption kinetics

The influence of reaction duration was assessed by conducting lead uptake experiments employing 200 mg/L of TMIP-206, a lead concentration of 100 mg/L, a sample volume of 100 mL, pH of 6, temperature of 25 °C, and reaction times spanning from 1 to 180 min. Following the separation of the adsorbent, the residual lead content was determined. Subsequently, the lead uptake performance of TMIP-206 was computed. The acquired data underwent analysis using pseudo-first order, pseudo-second order, and Elovich kinetic models. The corresponding fitting parameters were computed. The respective formulations for the kinetic models are detailed in Eqs. (6), (7) and (8).

6 $${q}_{t}={q}_{m}(1-{e}^{-{K}_{1}\times t})$$

7 $${q}_{t}=\frac{{K}_{2}\times {q}_{m}^{2}\times \text{t}}{1+{K}_{2}\times {q}_{m}\times \text{t}}$$

8 $${q}_{t}=\frac{1}{\upbeta }Ln\left(\alpha \beta \right)+\frac{1}{\upbeta }Ln\left(t\right)$$

In this context, q_t_ (mg/g) signifies the lead uptake performance at time t, q_m_ (mg/g) represents the theoretical lead uptake capacity, while K_1_ (1/min) and K_2_ (g/mg.min) are the respective fitting parameters for the kinetic equations. In the Elovich kinetic model, α (mg/g.min) denotes the initial adsorption rate, while β denotes the desorption constant.

### Methodology for pH study on lead adsorption

The impact of varying pH levels in water samples was explored through lead uptake experiments employing TMIP-206. These experiments included an application of 200 mg/L of TMIP-206, a pH range spanning from 2 to 8 and a Pb ion concentration of 100 mg/L. Adjustments to pH were achieved by 0.04 M HCl or NaOH. The residual lead content in each sample was quantified, and the lead uptake capacity was calculated using Eq. (1).

### Methodology for recovery study

For the regeneration of the adsorbent, it underwent a rinsing process using distilled water, followed by elution with a 1M NaOH solution to eliminate the adsorbed lead ions. TMIP-206 underwent repeated washing and elution cycles until the eluent no longer exhibited the presence of lead ions. Subsequently, the regenerated adsorbent was subjected to a drying procedure, rendering it ready for subsequent reuse.

### Methodology for characterization

Nitrogen gas adsorption isotherms were acquired with a Micromeritics TriStar II Plus gas adsorption apparatus, and the data underwent analysis through the BET technique. For XRD analysis, a Bruker D8 Advance diffractometer was employed, with operational parameters set within a 2° to 10° range, utilizing Cu K radiation at 30 mA and 40 kV. FTIR analysis was carried out by PerkinElmer spectrum Two spectrometer, employing a blend of KBr and TMIP-206.

## Results and discussion

### XRD

**Figure 1:**
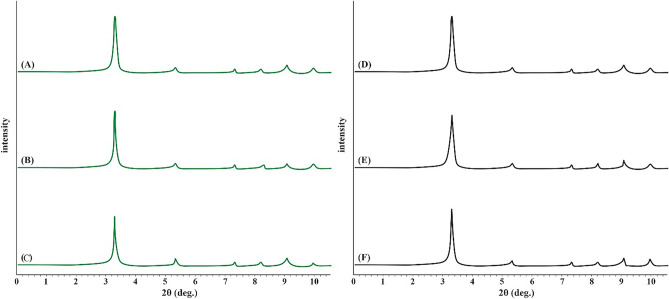
XRD spectrum of MIP-206 under water exposure (**A**) at day 0, (**B**) at day 15, (**C**) at day 30, and TMIP-206 under water exposure (**D**) at day 0, (**E**) at day 15, and (**F**) at day 30.

XRD analysis was performed to assess the crystallinity of TMIP-206 as well as water stability over a one month of water exposure. The presence of well-defined peaks in Fig. 1 signifies that TMIP-206 and MIP-206 exhibit a high level of crystallinity. The XRD pattern of TMIP-206 closely mirrors that of the original MIP-206, displaying peaks at 3.3°, 5.3°, 5.9°, 7.3°, 8.2°, 9.1°, and 10°, confirming the successful synthesis of MIP-206 in this study ^47^. This resemblance implies that the modification did not compromise the material's structural integrity or crystalline nature. Additionally, Fig. 1 illustrates that both MIP-206 and TMIP-206 demonstrated excellent water stability during one month of water contact as they maintained their crystalline structure almost intact.

### SEM

**Figure 2:**
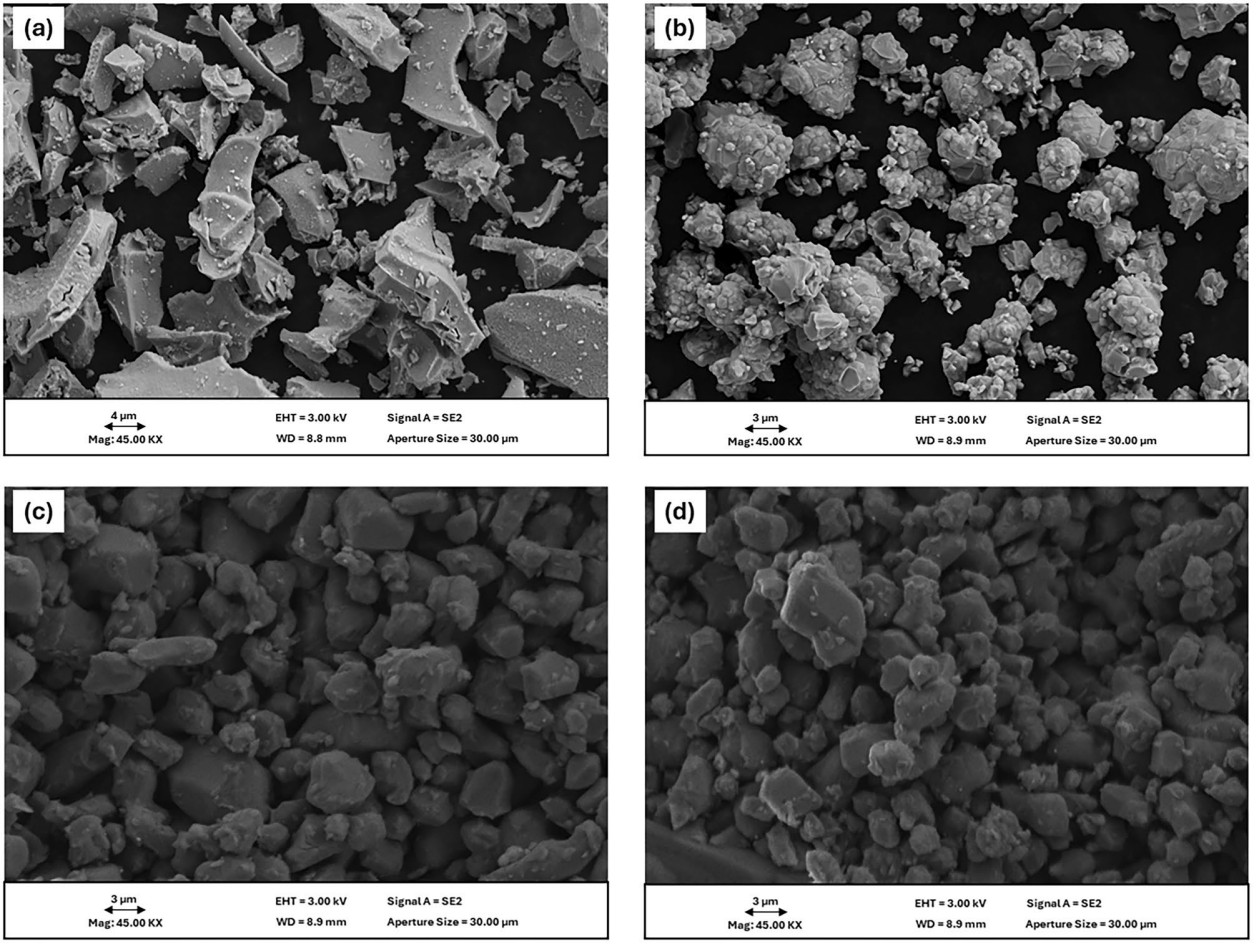
SEM images of MIP-206 (**a**) at day 0 of water exposure, (**c**) at day 30 of water exposure, TMIP-206 (**a**) at day 0 of water exposure, and (**b**) at day 30 of water exposure.

Figure 2 illustrates the SEM images of MIP-206 and TMIP-206. According to this figure, both MIPs exhibit crystals in irregular shapes with interconnected morphologies. The crystals have smooth surfaces with smaller crystal particles formed on the surface. Their crystal structure contributes to their stability and their interconnect morphologies add to their better mass transfer performance necessary for adsorption purposes. The size of the particles varies; however, most of the particles are in the range of 4–7 µm. After one month of water exposure both MIP-206 and TMIP-206 maintained their crystal structures, indicating their excellent stability under water even after one month which confirms the results obtained in XRD analysis.

### FTIR

**Figure 3:**
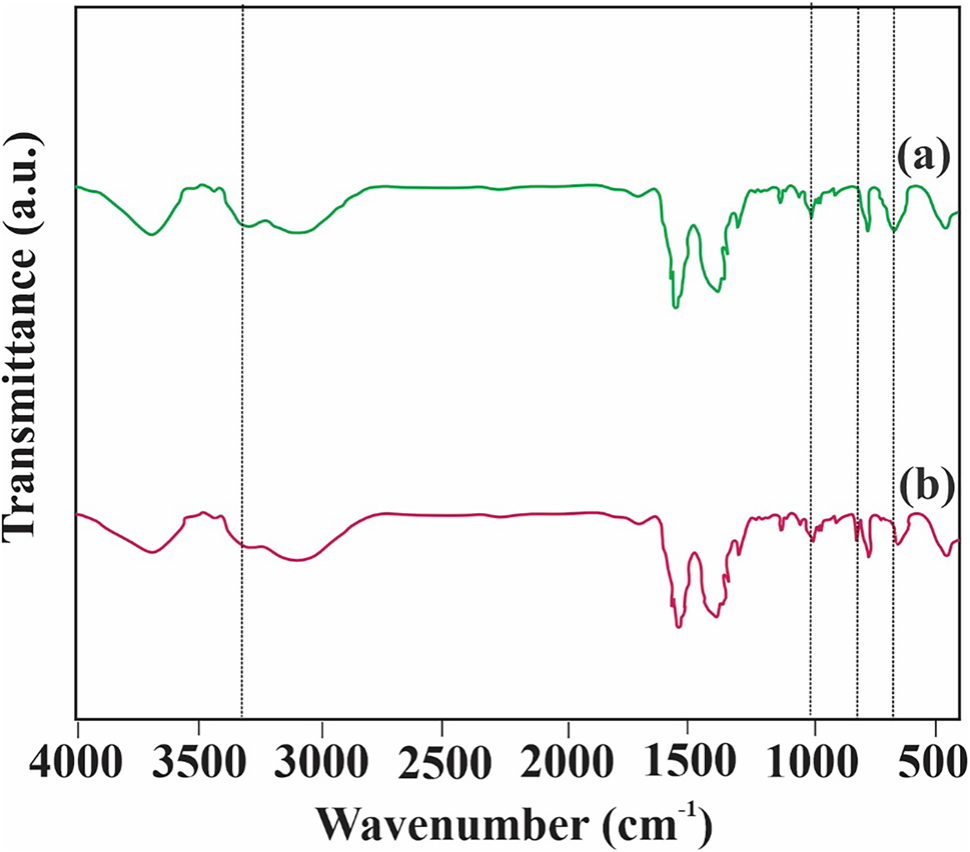
FTIR spectra of (**a**) TMIP-206, (**b**) TMIP-206 after adsorption.

The FTIR spectra depicting TMIP-206 can be observed in Fig. 3. In Fig. 3, TMIP-206 exhibited several absorption bands at specific wavenumbers, specifically 3301 cm^-1^ for (N–H), 2955 cm^-1^ for (C-H), 2847 cm^-1^ for (C-H), 1046 cm^-1^ for (C-N), and 660 cm^-1^ for (N–H) ^48,49^. Moreover, the robust presence of Zr-O bonds is signified by peaks at 669 cm^-1^ ^50^. The presence of nitrogen bonding characteristic peaks such as C-N and N–H indicates successful functionalization of MIP-206 with amine groups.

### TGA

**Figure 4:**
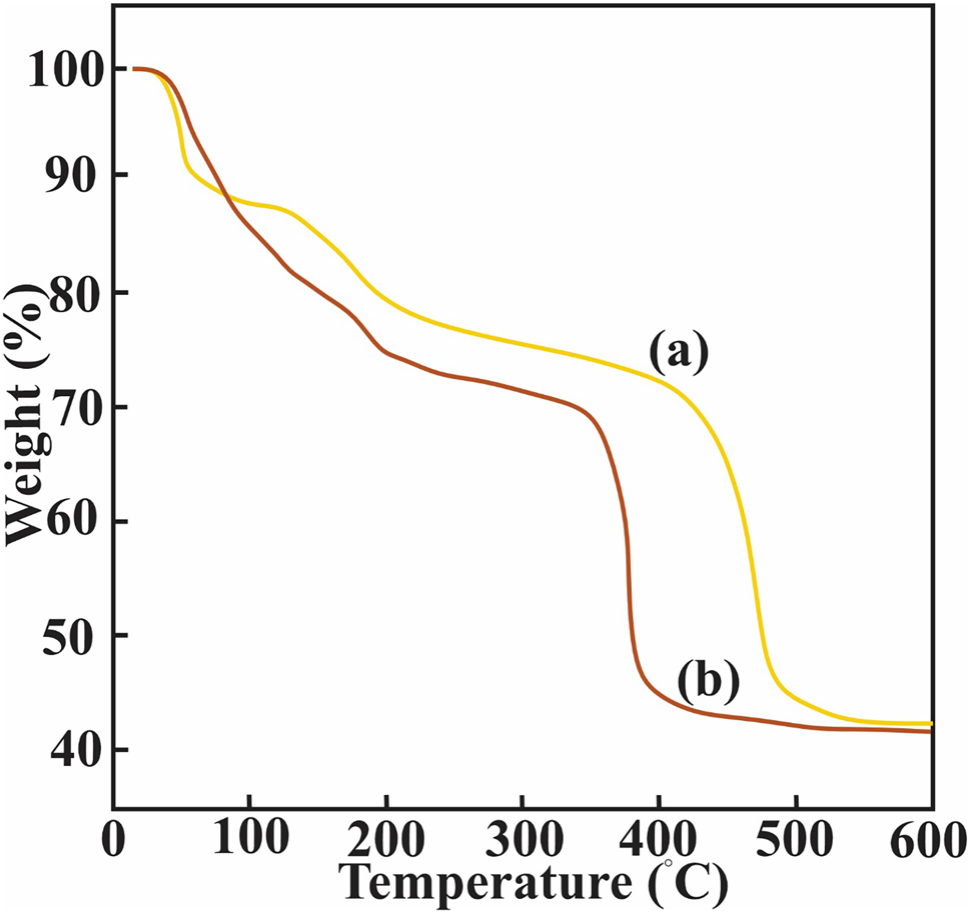
TGA curves: (**a**) MIP-206 and (**b**) TMIP-206.

The results of TGA analysis for MIP-206 and TMIP-206 are presented in Fig. 4. The weight reduction below 230 °C corresponds to the loss of water, trapped solvent, and the release of formates. However, there is an extra weight reduction step in TMIP-206 below 230 °C corresponding to the decomposition of triethylenetetramine moieties. The thermal response of MOFs to temperature increase starts at approximately 230 °C. The structural decomposition of MIPs corresponded to organic portion stops at 525 °C, at which the remaining weight is attributed to inorganic ZrO2 portion.

### BET

**Figure 5:**
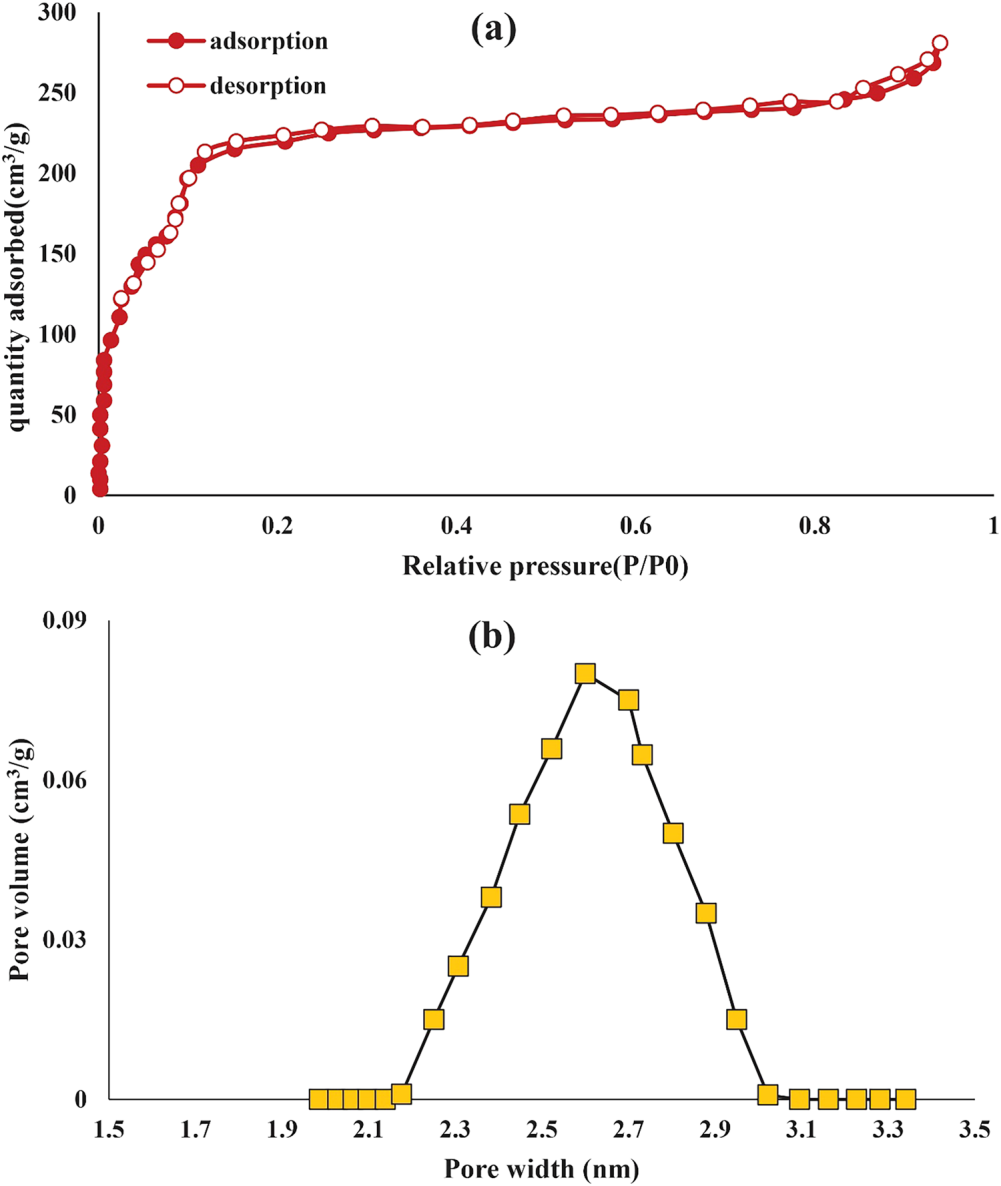
(**a**) N_2_ adsorption–desorption isotherm of TMIP-206. (**b**) Pore size distribution of TMIP-206.

Figure 5-a shows the nitrogen adsorption–desorption isotherm of TMIP-206. According to IUPAC classification, the nitrogen adsorption isotherm exhibits a type IV shape, indicating the presence of mesopores within its structure. As per the BET analysis, the determined surface area for TMIP-206 was 1015 m^2^/g (Fig. 5a). This notable specific surface area significantly facilitates the rapid adsorption of lead ions on the formulated adsorbent. In addition, the total pore volume of the TMIP-206 was found to be 0.41 cm^3^ g^−1^ (Fig. 5b). The pose size distribution of TMIP-206 indicates the presence of mesopores in the range of 2.1–3.3 nm, confirming the type IV isotherm type. The average pore size was observed to be 2.6 nm and Fig. 5-b shows a uniform size distribution.

### Lead adsorption isotherms and kinetics

**Figure 6:**
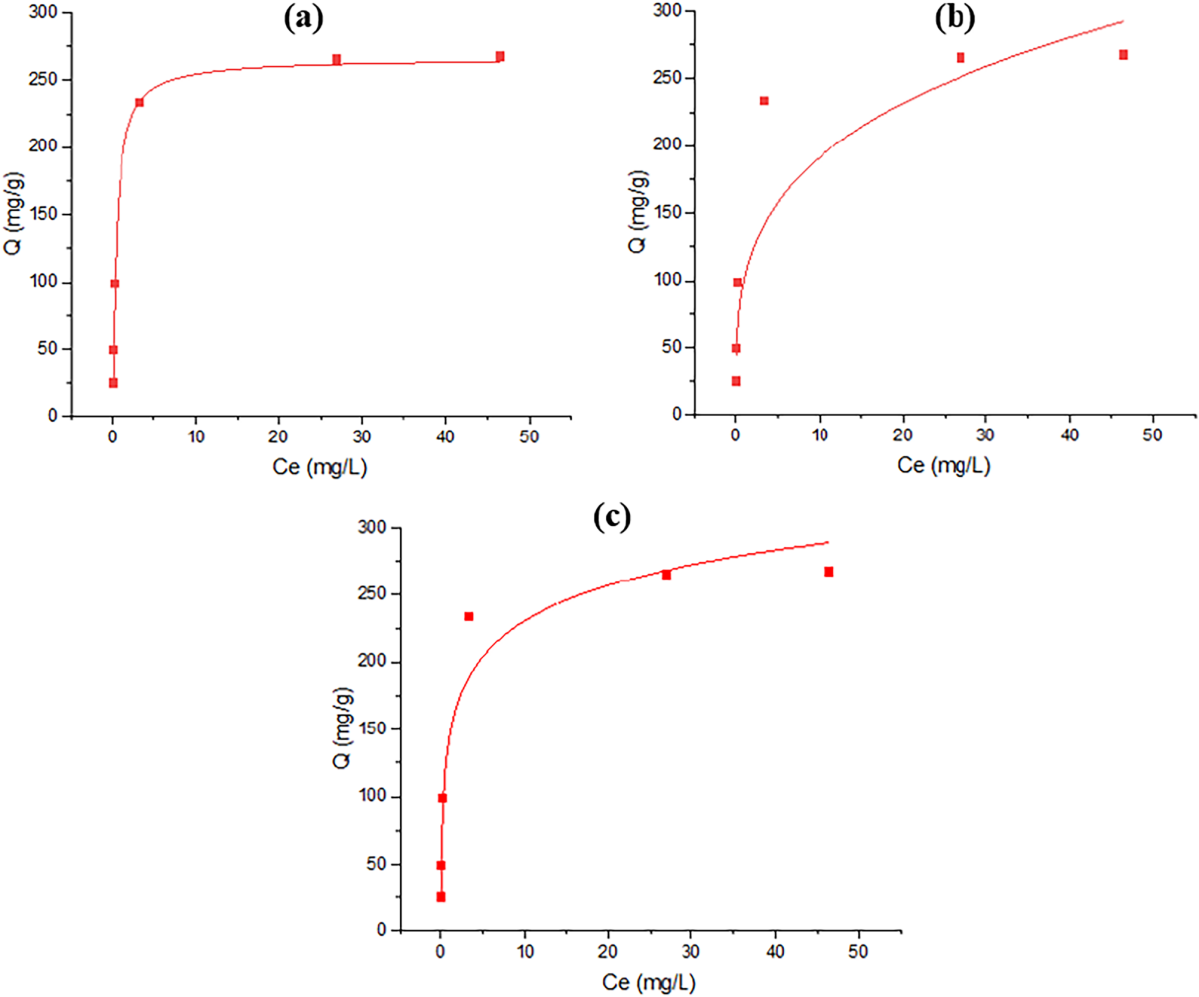
(**a**) Langmuir isotherm for TMIP-206, (**b**) Freundlich isotherm for TMIP-206, and (**c**) Temkin isotherm for TMIP-206**.**

**Figure 7:**
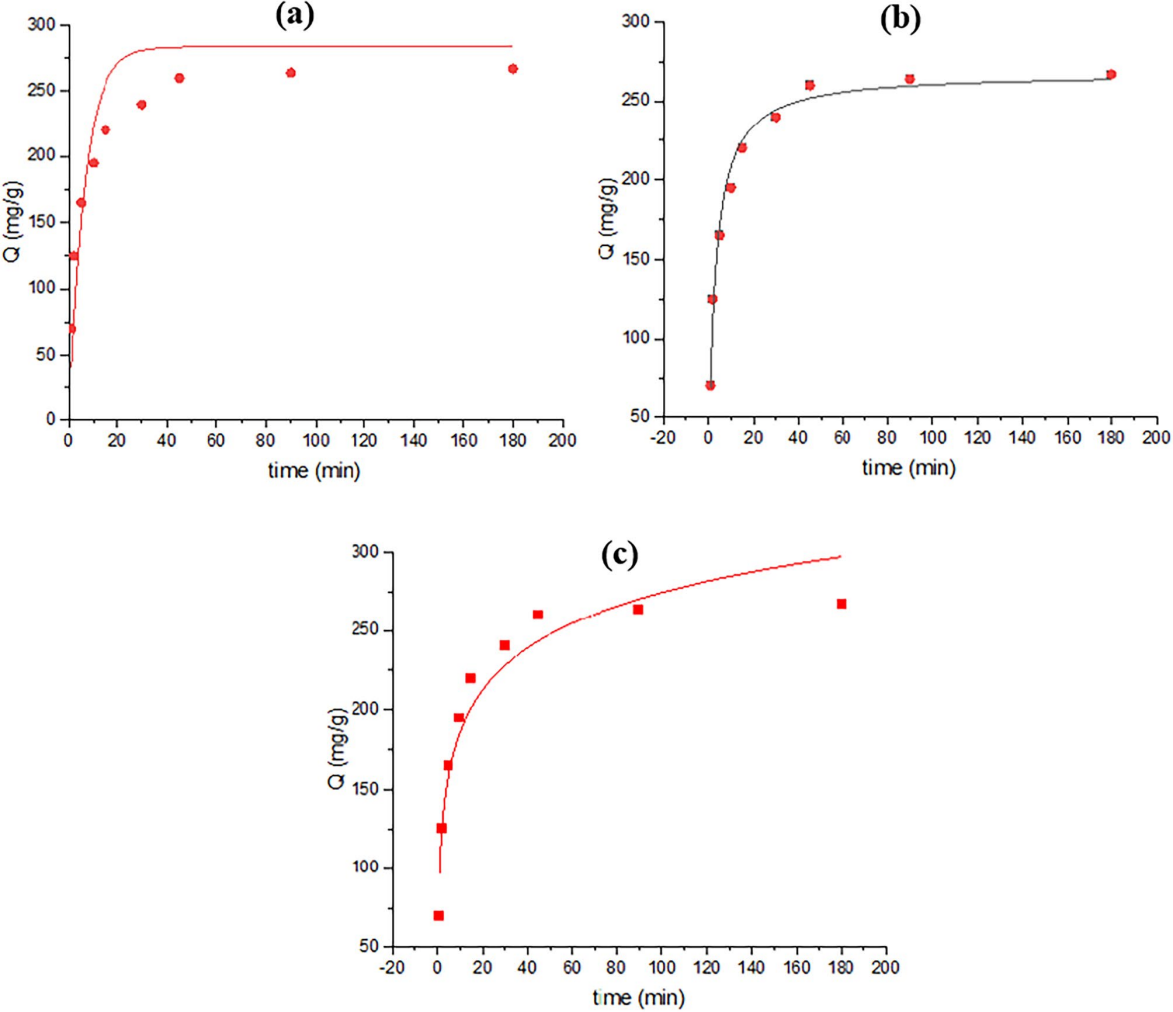
(**a**) Pseudo-first-order model for TMIP-206, (**b**) pseudo-second-order model for TMIP-206, and (**c**) Elovich model for TMIP-206.

**Figure 8:**
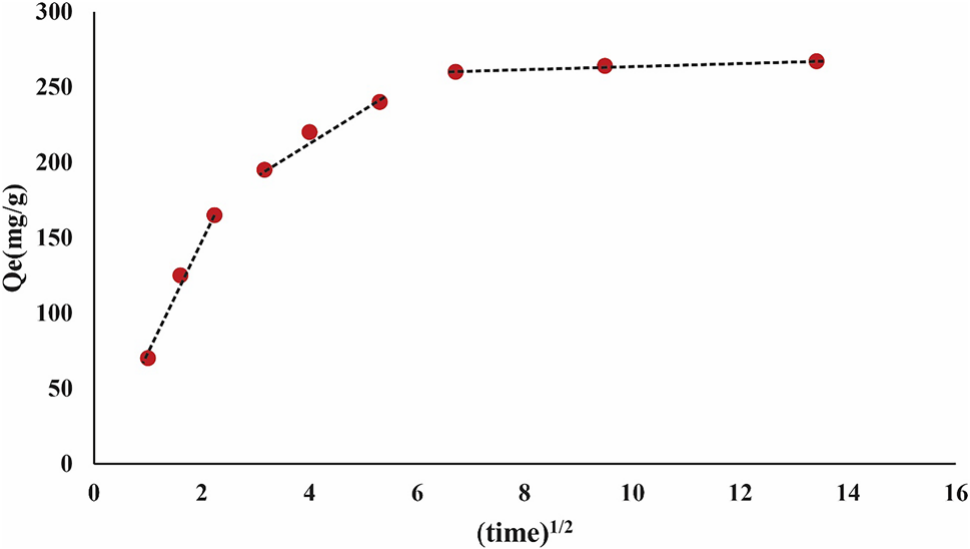
Intra-particle diffusion model for TMIP-206.

The current investigation aimed to elucidate the adsorption capabilities of TMIP-206, for lead removal. Langmuir, Freundlich, and Temkin isotherm equations were employed to analyze the kinetic data. The kinetic data was subjected to analysis by Elovich, pseudo-first, and pseudo-second-order models. The Langmuir isotherm determined the maximum theoretical removal capacity of Pb as 250 mg/g (Fig. 6 and Table 1). However, experimental results indicated actual Pb uptake of 267.15 mg/g, revealing a higher real lead sorption performance compared to the theoretical capacity. Figure 7 and Table 2 revealed that the lead adsorption behavior adhered to the pseudo-2nd-order model, suggesting chemisorption in the removal process. The Langmuir model demonstrated good agreement with the adsorption isotherms on TMIP-206, indicating uniform surface adsorption characterized by a restricted quantity of identical sorption sites. Findings suggest the significant potential of the synthesized MOFs as useful adsorbents for lead adsorption from wastewater. Table 3 presents the effectiveness of comparable adsorbents for Pb adsorption, showcasing the superior adsorption capacities of TMIP-206 compared to other reported values. These synthesized adsorbents exhibit remarkable adsorption capacities for lead elimination. Moreover, the intra-particle diffusion model was utilized to examine the kinetic data (Fig. 8). According to the intra-particle kinetic model, a linear graph intersecting the origin indicates that intra-particle diffusion is the only determining factor. Conversely, deviation from the origin on the graph implies the presence of additional processes influencing the rate, signifying that intra-particle diffusion alone does not govern the rate ^51,52^.

**Table 1:** parameters corresponding to isotherms for TMIP-206.

	Langmuir			Freundlich			Temkin		
	${\text{q}}_{\text{m}}$(mg/g)	${\text{K}}_{\text{l}}$(L/mg)	${\text{R}}^{2}$	$\text{n}$	${\text{K}}_{\text{f}}$(mg/g)	${\text{R}}^{2}$	A_T_ (L/g)	B_T_	R^2^
MIP-206	250	2.22	0.998	3.06	101.62	0.836	43.38	65.23	0.948

**Table 2:** parameters corresponding to kinetic models for TMIP-206.

Materials	Pseudo-first-order			Pseudo-second-order			Elovich		
	Theoretical ${\text{q}}_{\text{m}}$(mg g^-1^)	*K*_*1*_ (1/min)	*R*^*2*^	Theoretical ${\text{q}}_{\text{m}}$(mg g^-1^)	*K*_*2*_ (g/mg⋅min)	*R*^2^	*α* (mg/g.min)	*β*	*R*^2^
MIP-206	283.36	*0.16*	*0.782*	267.93	*0.0012*	*0.999*	*477.28*	*0.026*	*0.923*

**Table 3:** Comparison of Pb removal capacity of recently investigated MOFs.

MOFs	Lead ion removal capacity (mg/g)	References
Co-Al-LDH@CS/Fe_3_O_4_	558.84	^53^
UiO-66-NDC/GO	254.45	^54^
MIL-101	15.8	^55^
UiO-66-NHC(S)NHMe	49	^56^
NH_2_-SiO_2_@Cu-MOF	166.7	^44^
TMIP-206	267.15	Present study

### Adsorption mechanisms

The FTIR spectra following the adsorption of metal ions are depicted in Fig. 3b to investigate the mechanism underlying Pb ion removal by TMIP-206 and the associated functional groups. Our examination showed a slight decrease in the peaks at 660, 1046, and 3301 cm^−1^, pertaining to the bending vibration of the amine group, suggesting interaction between the amine group and lead ions. Additionally, a peak around 823 cm^−1^, potentially indicating the formation of N-Pb bonds, emerged post-lead ion removal, indicative of lead-amine coordination bonds formation.

In summary, the process of lead ion removal by TMIP-206 entails complex interactions between metal ions and amine functional groups.

### PH study on lead adsorption

**Figure 9:**
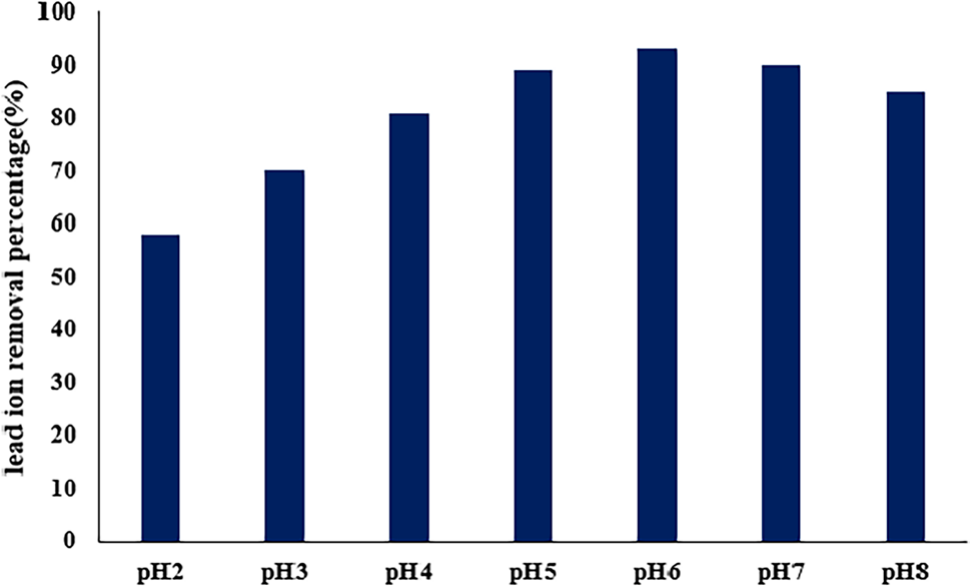
pH effect of TMIP-206 for lead ions adsorption process at C = 50 mg/L.

Prior research has demonstrated a significant influence of the initial solution pH on removal efficiency. Optimal adjustment of the pH not only minimizes matrix interference but also enhances the adsorption capacity to its maximum extent. The impact of pH was examined within a pH range of 2–8 at a lead concentration of 50 mg/L, and the outcomes are presented in Fig. 9. As indicated by the figure, the most effective pH for lead ion removal was determined to be 6. At lower pH values, an elevated concentration of hydrogen ions impedes the occupation of active adsorption sites by lead ions, resulting in a decrease in lead removal below pH 6. Conversely, with an increase in solution pH, the gradual creation of PbOH_2_ hinders the adsorption of Pb ion on the adsorbent, making it challenging to monitor their presence in that particular region.

#### Adsorbent reusability

**Figure 10:**
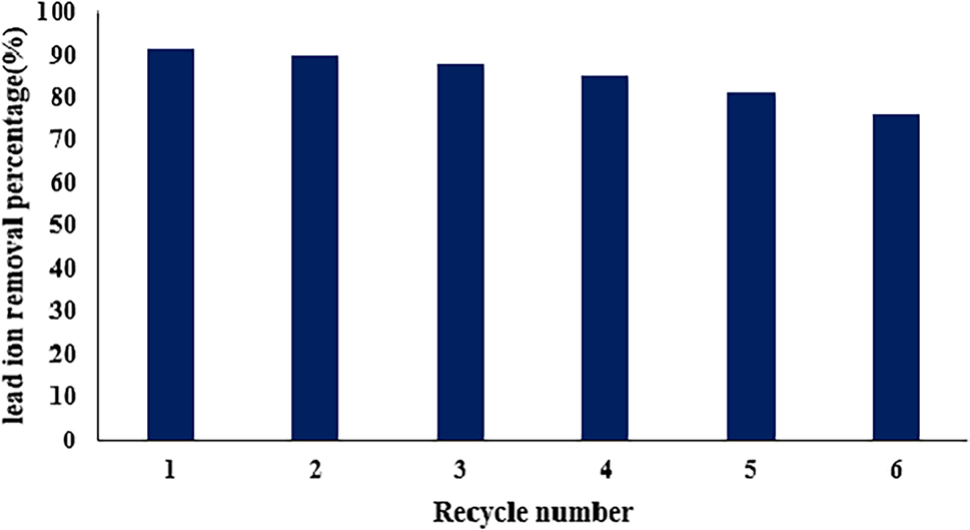
Reusability of TMIP-206 for lead ions adsorption process at C = 50 mg/L.

The regeneration of the adsorbent is a crucial aspect in in advancing a cost-effective adsorption process. The efficiency of Pb removal by the synthesized MOFs exhibited a slight decline with each successive of recycling sessions (Fig. 10). This fact can be ascribed to the gradual loss of pore volume during the successive adsorption–desorption cycles, wherein certain Pb particles may become entrapped within the adsorbent structure, impeding their complete removal even after regeneration. Despite this, the performance of the as-synthesized MOFs in Pb removal remained relatively stable, experiencing a reduction of less than 15% over the course of five cycles. The sustained effectiveness of the MOFs through multiple cycles of Pb adsorption and desorption underscores their durability and potential for long-term use.

### Study of the impact of interfering ions on Pb uptake using TMIP-206

The utilization of TMIP-206 for the treatment of a real wastewater sample was performed to examine its applicability for real-life scenarios. Table 4 presents the physicochemical characteristics of an actual wastewater sample rich in Pb^2+^ before and after the treatment process. TMIP-206 exhibited a high affinity for Pb^2+^ ions and reduced its content from 56.6 to 0.3 mg/L, indicating its excellent performance for the removal of Pb^2+^ ions. Additionally, some positively charged ions such as Cu^2+^ and Fe^2+^ were adsorbed on the surface of TMIP-206 due to similarity in charge and size. However, anions such as N${\text{O}}_{3}^{-}$, P${\text{O}}_{4}^{3-}$, and SO_4_^2−^ underwent negligible changes during the treatment due to repulsive forces from the negatively charged surface of TMIP-206.

**Table 4:** Physiochemical properties of real water sample before and after adsorption using TMIP-206.

Parameters	Before Adsorption with TMIP-206	After Adsorption with TMIP-206
Pb^2+^	56.6 mg L^−1^	11.2 mg L^−1^
Al^3+^	9.7 mg L^−1^	3.5 mg L^−1^
N${\text{O}}_{3}^{-}$	19.1 mg L^−1^	16.3 mg L^−1^
Mg^2+^	3.2 mg L^−1^	0.8 mg L^−1^
Cu^2+^	5.7 mg L^−1^	0.6 mg L^−1^
P${\text{O}}_{4}^{3-}$	16.4 mg L^−1^	13.1 mg L^−1^
Fe^2+^	19.2 mg L^−1^	7.2 mg L^−1^
SO_4_^2−^	7.6 mg L^−1^	6.5 mg L^−1^
${\text{Cl}}^{-}$	6.3 mg L^−1^	5.9 mg L^−1^

## Conclusion

In summary, a successful adsorbent was synthesized for the removal of Pb ions from water, utilizing TMIP-206. The isotherm analysis of lead adsorption revealed that the Langmuir model provides the most accurate description of the adsorption behavior. Notably, the Langmuir model yielded a maximum removal capacity of 267.15 mg/g, underscoring the remarkable efficiency of TMIP-206 in eliminating Pb ions from water. The initial pH value emerged as a critical influencing factor, with the optimum pH value identified as 6. The findings further highlighted the exceptional effectiveness of the adsorbent across a broad range of Pb(II) concentrations (5–100 mg/g) in test samples, reaffirming its efficacy under varied environmental conditions.

In conclusion, TMIP-206 crystals demonstrated substantial promise as an adsorbent for the efficient lead adsorption from aquatic environments. This research contributes valuable insights into the potential application of TMIP-206-based materials in addressing water pollution challenges, emphasizing their versatility and effectiveness in treating both low and high concentrations of lead contamination in water sources.

## Data Availability

All data generated or analyzed during this study are included in this published article.
